# Comparison of OBGYN postgraduate curricula and assessment methods between Canada and the Netherlands: an auto-ethnographic study

**DOI:** 10.3389/fmed.2024.1363222

**Published:** 2024-03-27

**Authors:** Emma Paternotte, Marja Dijksterhuis, Angelique Goverde, Hanna Ezzat, Fedde Scheele

**Affiliations:** ^1^Department of Obstetrics and Gynaecology, Gelre Hospitals, Apeldoorn, Netherlands; ^2^Department of Obstetrics and Gynaecology, Amphia Ziekenhuis, Breda, Netherlands; ^3^Department of Obstetrics and Gynaecology, University Medical Center Utrecht, Utrecht, Netherlands; ^4^Division of General Gynaecology and Obstetrics, University of British Columbia, Vancouver, BC, Canada; ^5^Department of Obstetrics and Gynaecology, Onze Lieve Vrouwe Gasthuis (OLVG), Amsterdam, Netherlands

**Keywords:** postgraduate medical education, assessment, EPA, comparison, auto-ethnographic

## Abstract

**Introduction:**

Although the Dutch and the Canadian postgraduate Obstetrics and Gynecology (OBGYN) medical education systems are similar in their foundations [programmatic assessment, competency based, involving CanMED roles and EPAs (entrustable professional activities)] and comparable in healthcare outcome, their program structures and assessment methods considerably differ.

**Materials and methods:**

We compared both countries' postgraduate educational blueprints and used an auto-ethnographic method to gain insight in the effects of training program structure and assessment methods on how trainees work. The research questions for this study are as follows: what are the differences in program structure and assessment program in Obstetrics and Gynecology postgraduate medical education in the Netherlands and Canada? And how does this impact the advancement to higher competency for the postgraduate trainee?

**Results:**

We found four main differences. The first two differences are the duration of training and the number of EPAs defined in the curricula. However, the most significant difference is the way EPAs are entrusted. In Canada, supervision is given regardless of EPA competence, whereas in the Netherlands, being competent means being entrusted, resulting in meaningful and practical independence in the workplace. Another difference is that Canadian OBGYN trainees have to pass a summative written and oral exit examination. This difference in the assessment program is largely explained by cultural and legal aspects of postgraduate training, leading to differences in licensing practice.

**Discussion:**

Despite the fact that programmatic assessment is the foundation for assessment in medical education in both Canada and the Netherlands, the significance of entrustment differs. Trainees struggle to differentiate between formative and summative assessments. The trainees experience both formative and summative forms of assessment as a judgement of their competence and progress. Based on this auto-ethnographic study, the potential for further harmonization of the OBGYN PGME in Canada and the Netherlands remains limited.

## Introduction

It is widely acknowledged that well-structured postgraduate medical training is important to deliver well-trained medical specialists who can provide the best possible care. To certify that trainees develop the necessary skills and attitudes in addition to the knowledge required to practice, the competency framework CanMEDS was developed in Canada in 1996 (1). This competency-based framework described the competences that trainees should acquire during their training. In 2015, the CanMEDS framework was enhanced by incorporating competency milestones for each CanMED role (2).^1^ This approach has been implemented in several medical education systems globally. For instance, the CanMEDS roles have been used in the official training blueprints of all postgraduate medical education (PGME) in the Netherlands (3).^2^

Further expansion of the CanMEDS has led to the development of EPAs. EPAs are described as Entrustable Professional Activities. The Netherlands was among the pioneers in implementing EPAs in their training programs (4–8). The Obstetrics and Gynecology PGME training in Canada also transitioned toward EPAs (see text footnote 1). EPAs are “units of professional practice, defined as tasks or responsibilities to be entrusted to unsupervised execution by a trainee once he or she has attained sufficient competence” (9).

The EPAs are designed to ensure a gradual level of entrustment, known as progressive independence. Entrustment is intended to mean trust, meaning that if the postgraduate trainee is entrusted to perform a certain activity, they would be expected to carry out the procedure without supervision (4). For instance, potential applications of EPAs include assessing competence, making entrustment decisions for independent practice, facilitating professional development, and informing curriculum development. It is known that the uptake of EPAs in the curricula varies among the different specialty training programs (10).

Although competency-based training, CanMeds roles, and EPAs have been implemented in postgraduate OBGYN-training in several countries, assessment methods considerably differ. Differences can be attributed to political, social, and cultural reasons (4, 11). It has been suggested that patient outcomes, notably major complications, are associated with the quality of training received. Interestingly, quality of training is not associated with licensing examination scores (12). Aabake et al. demonstrated that in Europe, examinations during OBGYN training were used in 89% of the included countries. All examinations were mandatory, but the study did not clarify whether they were formative or summative (11). In other words, formative assessments are often considered assessments *for* learning, while summative assessments are assessments *of* learning (13). It is interesting to find out why some postgraduate OBGYN training programs use certain assessment tools, and why others do not. For instance, despite Canada having implemented competency-based training and EPAs, the adoption of new assessment methods and the utilization of a training logbook are not yet widespread practices (14). As stated on the Royal College website,^3^ new formal assessment methods have been implemented and formalized since Garofalo's findings in 2017.

Worldwide, the Netherlands and Canada hold leading positions in the advancements and modernization of medical education (14). Given the similar foundations of PGME in Canada and the Netherlands, it is interesting to discuss differences, particularly in competency-based education and programmatic assessment. Therefore, our research questions are “Which differences in formal assessment methods exist in Obstetrics and Gynecology postgraduate medical education and how does this impact the advancement to higher competency for the postgraduate trainee?” To answer these questions, we employed the auto-ethnographic observation method to gain insights in which differences exist and how these differences are perceived by the postgraduate trainees. We scrutinized the Obstetrics and Gynecology PGME blueprints of both countries to find similarities and differences. Highlighting the differences between both Canadian and the Dutch assessments systems with respect to cultural and political differences may provide insight into assessments that could enhance each training program. By harmonizing curricula, variability in training outcomes will decrease, which could improve the quality of training overall. Moreover, it would facilitate benchmarking and comparisons across centers and streamline the exchange of trainees between countries (14).

## Materials and methods

This study is grounded in auto-ethnography, a qualitative research methodology in which the author uses writing and self-reflection to probe personal experiences, thereby deriving broader sociocultural meaning and understanding (15).

Auto-ethnography combines elements from autobiography and ethnography and represents both a scholarly “process” and “product.” It moves away from traditional approaches to understanding and representing “culture” which has historically been rooted in rigid structures. Instead, auto-ethnography “acknowledges and accommodates subjectivity, emotionality, and the researcher's influence on research, rather than hiding from these matters or assuming they don't exist” (16).

### Participants and setting

This is qualitative research includes an introduction of the enrolled researchers. This project involved five participants: EP, FS, MD, AG, and HE. The main auto-ethnographer was EP, a Dutch 6th-year postgraduate trainee in OBGYN who finished an elective in Vancouver, Canada. She is the subject of the performed auto-ethnographic study and received support and supervision throughout the project from the other four participants. FS is gynecologist and professor in Health Systems Innovation and Education in the Netherlands, where he supervised PhD students and conducted research in assessment methods. MD is a gynecologist in the Netherlands with a PhD in assessment methods in medical education. AG is gynecologist and program director of EP in postgraduate medical education of OBGYN in Utrecht, the Netherlands. HE is gynecologist and program director of the postgraduate residency program in British Colombia, Canada. The connection among our team members was forged serendipitously.

### Data collection

The predetermined guiding theme for this auto-ethnography was how postgraduate trainees perceive assessment methods and how formal assessment methods are shaped throughout the course of residency training. EP's experiences as an elective OBGYN postgraduate trainee were discussed with the other researchers. As the process of reflection and discussion developed, the need to scrutinize the blueprints of PGME of Canada and the Netherlands became apparent. Differences and similarities were found. These findings were then discussed with gynecologists and postgraduate trainees of both the Netherlands and British Columbia, Canada. Extensive reflection and discussion between EP and these trainees and gynecologists yielded insights into how postgraduate trainees perceive their assessment methods and how does this impact the advancement toward higher competency. These reflective sessions were either audiotaped or noted.

## Results

The results are presented in two sections. The first section provides a general overview based on the blueprints of the Canadian and the Dutch curricula, with a focus on assessment methods. In Table 1, an overview of postgraduate medical education in OBGYN in both Canada and the Netherlands is given. Table 2 describes the EPAs in the Netherlands, whereas Table 3 describes the EPAs in Canada.

**Table 1:** Overview of PGME in OBGYN focused on assessment.

	**Canada**	**The Netherlands**
Years of UGME/Med school	4	6
Years of PGME OBGYN	5	6
EPAs in PGME OBGYN	36	12
Working hours per week	No duty hour restrictions	Maximum 48 h
Feedback	Formative throughout the whole year	Formative throughout the whole year
Progress assessment	Every 6 months by competency committee	In the first year every 3 months, after first year at least annually, progress meeting by trainee and program director
Progress test	Yearly, formative, not required by Royal College	Yearly, formative, mandatory
Surgical examination	Year 2, summative	Mandatory courses
Final examination	Written and Oral	None
Postgraduate training program paid by:	Ministry of health. Money to cover resident salaries not given directly to hospitals, but to health authorities or larger bargain units. No funding for “disutility” provided.	Ministry of health, welfare and sports, executed by the Dutch healthcare Authority (NZA). Money is forwarded to the teaching hospital where the trainee is currently employed. It covers both costs for training (salary and education) and disutility.

**Table 2:** Overview of EPAs of OBGYN in the Netherlands (17).

Core EPA # 1: Pregnancy and labor, low-risk
Core EPA # 2: Pregnancy and labor, high-risk
Core EPA # 3: Postpartum healthcare
Core EPA # 4: Abnormal uterine bleeding
Core EPA # 5: Early pregnancy
Core EPA # 6: Prolapse and pelvic floor complaints
Core EPA # 7: Abdominal pain (acute and chronic)
Core EPA # 8: Vulvar and vaginal abnormalities
Core EPA # 9: Healthcare for pre-malignant lesions
Core EPA # 10: Basic oncological care
Core EPA # 11: Fertility healthcare
Core EPA # 12: Lifecycle endocrinology

**Table 3:** Overview of EPAs of OBGYN in Canada^*^ (see text footnote 4).

Transition to Discipline EPA #1: Performing initial assessments for uncomplicated obstetric patients.
Transition to Discipline EPA #2: Performing initial assessments for uncomplicated gynecological patients.
Foundations EPA #1: Providing routine prenatal care to a low-risk, healthy population.
Foundations EPA #2: Performing assessments of antenatal fetal well-being.
Foundations EPA #3: Assessing and providing initial management for patients with common obstetric presentations.
Foundations EPA #4: Managing labor and childbirth.
Foundations EPA #5: Performing uncomplicated cesarean sections with a skilled assistant.
Foundations EPA #6: Providing early postpartum care.
Foundations EPA #7: Providing consultation and initial management for patients with urgent and emergent gynecologic presentations.
Foundations EPA #8: Counseling and management for patients requiring family planning.
Foundations EPA #9: Providing consultation for patients with gynecological conditions.
Foundations EPA #10: Performing minor gynecologic operative procedures.
Foundations Special Assessment #1: Performing critical appraisal of health literature and initiating scholarly projects.
Core EPA #1: Providing preconception and antenatal care to women with high-risk pregnancies.
Core EPA #2: Managing patients with acute conditions presenting in the antenatal and perinatal periods.
Core EPA #3: Managing complex vaginal deliveries.
Core EPA #4: Performing complex cesarean sections.
Core EPA #5: Diagnosing and managing postpartum complications.
Core EPA #6: Performing obstetric and gynecologic ultrasound.
Core EPA #7: Providing definitive management for patients with acute gynecologic emergencies.
Core EPA #8: Providing care for patients with complex gynecologic conditions and/or medical comorbidities
Core EPA #9: Assessing and initiating management for patients with reproductive challenges
Core EPA #10: Diagnosing and managing pediatric and adolescent patients with common gynecologic conditions.
Core EPA #11: Providing care for patients with pelvic floor dysfunction.
Core EPA #12: Assessing, diagnosing, and managing patients with chronic pelvic pain and sexual health concerns.
Core EPA #13: Assessing and managing patients with gynecologic malignancies.
Core EPA #14: Performing advanced hysteroscopy.
Core EPA #15: Performing major vaginal and vulvar procedures.
Core EPA #16: Performing major laparoscopic gynecologic procedures.
Core EPA #17: Performing major open abdominal gynecologic procedures.
Core EPA #18: Managing patients with surgical complications.
Core EPA #19: Managing the birthing unit.
Transition to Practice EPA #1: Managing complex patients, including those requiring longitudinal care.
Transition to Practice EPA #2: Discussing difficult news.
Transition to Practice SA #1: Conducting scholarly work.
Transition to Practice SA #2: Teaching and managing learners.

^*^The Canadian system of EPAs is subdivided into stages. Stage 1 is transition to discipline, stage 2 is foundations, stage 3 is core, and stage 4 transition to practice. This structure is maintained across all postgraduate programs in all disciplines. The trainee must obtain all the EPAs for a certain stage before the competency committee promotes them to the next stage, and they can start working on the associated EPAs for that stage.

The second part is a discussion of how these assessment methods manifest in the workplace. This discussion is based on the experiences of EP and is the auto-ethnographic part.

### PGME and assessment of OBGYN in the Netherlands

Postgraduate training can be pursued directly after 6 years of undergraduate medical training. Typically, candidates undergo a period of work in non-training grade posts in order for the candidate to gain practical experience and increase their chances of successfully applying for a postgraduate training program. Entering a postgraduate training program is based on an interview, which may sometimes be combined with a personal assessment. The postgraduate training program in OBGYN lasts 6 years on full-time basis, although part-time placement is allowed to a certain degree. The program consists of hands-on practical training in delivering patient care, complemented by formal teaching, and academic study. Programs are completed in a structured learning environment in teaching hospitals supervised by faculty. Rotations are evenly divided between academic and district teaching hospitals.

The national training curriculum is based on EPAs (Table 2), and progress is registered in the personal electronic portfolio. The curriculum focusses on delivering competent general OBGYNs who share common basic skills but have specific areas of differentiation, depending on their area of interest. The curriculum of the OBGYN postgraduate training is captured in a national training curriculum for obstetrics and gynecology LOGO (Landelijk Opleidingsplan Obstetrie en Gynaecologie) (17), where a daily feedback culture is a basic requirement for the programs. One of the most salient features of current training is the increased number and variety of formative assessment moments: assessments that are aimed at providing feedback that can direct and stimulate learning. Additionally, an annual formative online progress test, which consists of 160 multiple choice questions, is mandatory. This test is designed to generate learning goals based on the test results. The low-stakes formative assessments and learner self-reflection are pivotal to progression within the training program and not the formative annual national written progress test (8). There is no final oral or written examination. Moreover, postgraduate trainees may participate in mandatory or non-mandatory courses, such as an ultrasound course. All formative assessments, together with progress reports, proof of experience, feedback of supervisors [for example Objective structured assessment of technical competencies (OSATS) and mini clinical evaluation exercise (mini-CEX)], individual reflection, and the results of the annually formative progress test are collected in a personal electronic portfolio.

Assessment for entrustment of specific EPAs and the final conclusion on whether or not the postgraduate trainee is competent to deliver high standards of care is conducted by the educational team chaired by the program director and is based on the records in the electronic portfolio (all activities and development). If the EPA is entrusted, the trainee is allowed to perform this EPA independently.

### PGME and assessment of OBGYN in Canada

Medical school students seeking to start postgraduate training in OBGYN apply to the Canadian residency matching program. This residency matching program includes a detailed portfolio, and the selection process includes a file review of medical school performance records, reference letters, and an interview. The specialty training requirements in OBGYN include a 5-year training program governed by the Royal College of Physicians and Surgeons of Canada. A new curriculum, called Competency by Design, was introduced in 2019 (16).^4^ The curriculum is framed around 36 EPAs (Table 3).^5^ These EPAs represent essential specialist competencies, leading to optimal OBGYN care outcomes. The EPAs are designed to align with the stage the trainee is in. The pedagogical concept is that EPAs should not be time based, but based on whenever a competency has been demonstrated. However, as has been stated in *The Future of medical education in Canada*^6^: “this approach to using competencies to assess the performance of physicians in practice is still in its infancy.”

Currently, the assessment philosophy of Canadian medical education is based on this competency by the design structure. This philosophy includes formative assessments throughout the whole year. The workplace-based assessments include the following, for instance: OSATS, mini-CEX, Case-based discussion (CbD), Team observation forms, bi-Annual Review of Competence Progression by the competency committee, and Non-Technical Skills for Surgeons (NOTSS). The concept is that continuous assessment drives and promotes learning. In year 2, the postgraduate trainee of surgical specialties, such as OBGYN, needs to pass a summative surgical foundations examination examination (see text footnote 3). In year 5, a summative written examination is taken, followed by the final summative oral examination 6 months later. If the postgraduate trainee fails one of these summative examinations, the trainee is required to retake the failed examinations. Additionally, there is an annual formative written progress test, which is not mandatory but is utilized to prepare trainees for the final examination (see text footnote 1) (14).

The formal methods of assessment are described in the “Standards of Accreditation for Residency Programs in Obstetrics and Gynecology” (see text footnote 1). The competency committee comprises supervisors (gynecologists) from various clinics, as well as the program director. Together, they decide whether an EPA is entrusted. Even after an EPA is entrusted, some degree of ongoing direct or indirect supervision remains the cultural, legal, and practically enforced norm.

### Reflection

Postgraduate medical education in Canada and the Netherlands share the same philosophy, including programmatic assessment in competency based medical education. Medical education in Canada and the Netherlands are regarded as the highest standards worldwide (13). In both countries, development and improvement are key factors in medical education.

In both the Netherlands and Canada, EPAs and programmatic assessment have been implemented successfully and this has been confirmed by stakeholders. Despite these similarities, there are a few key differences in the curricula and assessment methods experienced by EP and discussed with the research team.

**The first** difference between OBGYN education is the total duration of medical training. In Canada, medical school (undergraduate training) and residency (postgraduate medical training) embrace a total of 9 years, whereas in the Netherlands, total duration is 12 years, usually longer due to junior doctors commonly working in non-training posts. Another key finding that aligns with the first difference is that non-training posts are not possible in Canada. In Canada, non-training posts for junior doctors who have finished medical school do not exist.

**The second** difference is the total amount EPAs. In the Netherlands, the 12 EPAs are used for the whole 6 years of postgraduate training, while in Canada, EPAs are organized into stages. The stages represent a stepwise progression of competence during the training period. Comparing the content of the EPAs informs us that there are no big differences in the end terms of the OBGYN training. However, it appears that the Canadian OBGYN EPAs are more fragmented than those in the Netherlands.

**The third** difference lies in how entrustment is applied by both countries. In the Netherlands, OBGYN has implemented this entrustment process in daily clinical practices; a trainee, entrusted to perform a cesarean section, is legally and culturally allowed to perform the procedure unsupervised. For the trainee, the entrustment granted during the training years contributes to an inner sense of confidence that they indeed are capable of working as a gynecologist (unsupervised). Most PGME programs in the Netherlands offer the trainees with rotations where they can experience the role of supervising younger trainees, which contributes to their confidence and professional growth toward independent practice. In the Netherlands, entrustment translates into a meaningful change in status. In contrast, in Canada, entrustment means that the resident is progressing as would be expected for their training. Canadian trainees are usually supervised during (surgical) procedures until their certification as a gynecologist. Both postgraduate trainees and supervisors describe this as the norm. A quote from one of the supervisors is as follows: “Competency by design is still a bit out of reality of daily clinical education.”

An explanation for this significant difference in the meaning of entrustment might be found in how the levels of entrustment are interpreted. As explained on the website of the Royal College, the steps of assessment to full entrustment of an EPA are described by the use of the O-score. This score is a 9-item evaluation tool designed to assess technical competence in surgical trainees using behavioral anchors. This O-score seems to be written through the eyes of the supervisor (highest level: “I did not need to be there”), whereas in the Netherlands, this scoring system is through the eyes of the trainee (highest level: “I supervise this procedure or EPA”). The entrustment rating of an EPA by the Royal College is as follows: “Rating trainees as independent does not mean that they are now always allowed to independently perform that task. It means that they were independent on this occasion” (18). The entrustment rating of independence does not reflect the medico-legal reality, nor the expectations of patients and trainers of the presence of a certified specialist who is ultimately responsible, especially in surgery. The reason for this may be found in how the regulation and granting of postgraduate medical education is arranged in Canada as the Royal College is responsible for prescribing and assessing the learning standard, but not for regulating or granting a license to practice. Ultimately, all trainees, even until the last moment of training, have an educational license only, which means that they have no license to practice independently and everything they do must be supervised. The supervisor is the “most responsible physician” if something goes wrong. It is understandable why a faculty surgeon, who owns that responsibility, might hesitate to allow a resident, even one they believe is highly competent, to perform a cesarean section without supervision just because the competency committee (CC) said that they were competent to do so. However, the designation by the CC holds little weight outside the program.

**A fourth** difference is the formative and summative examination systems. Both countries have a yearly progress test, in the form of a written examination. This is obligatory for the Dutch resident, and its results are used formatively to identify areas for further study. However, in Canada, the yearly formative test is not mandatory; the Royal College does not require, endorse, or even suggest this test. Instead, the program directors and teachers want trainees to take the test as a necessity to prepare trainees for their final examination. This final examination has to be taken by postgraduate trainees in Canada but is not known in the Dutch postgraduate training program. Though competency by design in Canada preaches programmatic training of competencies and formative assessments, the final assessment is summative. Postgraduate trainees in Canada find it inconceivable to not have a final examination. The trainees described that supervisors treat them differently as soon as they passed this examination. Ending the PGME without this final examination is unimaginable for them, which gives them the feeling that they have met a certain standard and that this will allow them to get a job anywhere else in Canada. Legally the resident still does not have a license after passing the examination to act independently, but the examination certification is the key requirement to that license. In contrast, Dutch postgraduate trainees are satisfied with their assessment system without a summative exit examination, mainly because the Dutch trainee work totally independent at the end of their training and, therefore, a summative exit examination would not add anything to the growing process. Growing into being fully entrusted is an organic process that develops along the way. Dutch trainees view examinations as a snapshot of their performance and query how well any such examination would represent their competence. Interestingly, despite the formative intent of PGME in OBGYN in both countries, neither set of postgraduate trainees perceive their training as such. Many postgraduate trainees regard all the feedback moments as small summative assessments and feel continuously judged about their performance.

## Discussion

To answer our first research questions (“which differences in formal assessment methods exist in Obstetrics and Gynecology of postgraduate medical education?”), we created an overview of the formal assessment methods. For the second research question (“how does this impact the advancement to higher competency for the postgraduate trainee?”), we observed four main differences in the curricula of PGME of OBGYN between Canada and the Netherlands. The most striking difference lies in the way that entrustment is interpreted and put into practice in both countries, since this is of consequence for the role of assessment in the entrustment process and even more trainees feeling adequately prepared to work as a gynecologist. However, the Royal College explains the entrustment of EPAs as the resident is progressing as would be expected for their stage, for the Dutch trainees entrustment reflects their professional development toward independent practice. Additionally, the number of EPAs differs enormously, which might also reflect the difference between wanting control and needing a “pass” on smaller parts, vs. believing in trust the knowledge that the whole is more than the total sum of small parts. In addition to this consideration, there is the requirement for passing a summative exit examination in Canada, whereas in the Netherlands, this requirement has been rendered redundant. In conclusion, a higher competence is reached more or less on a similar way in both Canada and the Netherlands. However, the meaning of this higher competence is interpreted differently.

To summarize, programmatic assessment in Canada appears to be based on the “assessment *for* learning” principle. The fact that there is a summative final examination also makes assessment feel more like an “assessment *of* learning” or perhaps it is a combination of the two. In contrast, programmatic assessment in the Netherlands tends more toward “assessment for learning” alone. However, in both countries, trainees tend to experience the assessments as more summative than formative, in general, and there is ongoing discussion about how to make assessments feel formative for postgraduate trainees. We propose that there is a role for both kinds of assessment, in line with the different levels of knowledge and skills as described in Miller's pyramid (19).

Formative assessment is the instrument for coaching a trainee from the “does” level in Miller's pyramid to the “shows” level, for instance in surgical procedures. Summative assessment is the instrument to actually ascertain and document whether the trainee has reached the higher “shows” level and to inform supervisor and trainee about the level of independence in specific tasks or procedures. Making the aim of the formative assessment explicit could help the trainee to focus more on the learning process, while clearly laying out the summative assessment moments gives the trainee a perspective on entrustment decisions (20).

## Conclusion

There is no one-size-fits-all solution, and no specific programmatic assessment has been proven to be superior to others (21). A tip for both countries regarding their assessment methods is that clear communication about the purpose of assessment and the use of assessment outcomes needs to be formulated (22). All supervisors need to be trained to provide non-judgmental constructive formative feedback (20). Competency-based medical education requires an ongoing process to evaluate and improve the assessment methods (13). This difference in assessment program is largely explained by cultural and legal aspects of postgraduate training and consequent differences in licensing practice. Based on this auto-ethnographic study, the harmonization of the OBGYN PGME in Canada and the Netherlands appears limited since cultural, legal, and practical aspects of assessment and licensing predominate. In our opinion, this is a missed opportunity because harmonization could help to resolve labor shortages where needed. We recommend investigating whether there are options to facilitate the harmonization process between postgraduate trainees between the Netherlands and Canada.

## Limitations

A limitation of this study is that not all perspectives could be captured. There may be opinions and experiences of trainees, teachers, and supervisors that were missed. Other limitations include the inherent subjectivity of the ethnography method and the limited sample size.

## Data availability statement

The raw data supporting the conclusions of this article will be made available by the authors, without undue reservation.

## Ethics statement

Ethical approval was not required for the studies involving humans because it is an auto ethnographic study which does not contain personal information but only interpretations of the writer based on humans. The studies were conducted in accordance with the local legislation and institutional requirements. Written informed consent for participation was not required from the participants or the participants' legal guardians/next of kin in accordance with the national legislation and institutional requirements because no direct material of humans was used.

## Author contributions

EP: Conceptualization, Data curation, Formal analysis, Methodology, Project administration, Writing – original draft, Investigation. MD: Conceptualization, Supervision, Writing – review & editing. AG: Supervision, Writing – review & editing. HE: Writing – review & editing, Data curation, Methodology. FS: Writing – review & editing, Data curation, Methodology, Conceptualization, Supervision.

## Footnotes

^1^ https://www.royalcollege.ca

^2^ https://www.knmg.nl/ik-ben-arts/cgs/regelgeving/specialismen

^3^ https://www.royalcollege.ca/en/credentials-exams/assessment-canadian-us.html

^4^ https://www.royalcollege.ca/en/cbd/understanding-cbd.html

^5^ https://www.royalcollege.ca/content/dam/documents/accreditation/competence-by-design/non-resource-documents/epa-observation-templates-e.html

^6^ https://www.afmc.ca/wp-content/uploads/2022/10/FMEC-CPD_Synthesized_EN_WEB.pdf
